# Interleukin (IL)-33 is dispensable for *Schistosoma mansoni* worm maturation and the maintenance of egg-induced pathology in intestines of infected mice

**DOI:** 10.1186/s13071-020-04561-w

**Published:** 2021-01-22

**Authors:** Jean Pierre Kambala Mukendi, Risa Nakamura, Satoshi Uematsu, Shinjiro Hamano

**Affiliations:** 1 Program for Nurturing Global Leaders in Tropical and Emerging Communicable Diseases, Graduate School of Biomedical Sciences, Nagasaki University, Nagasaki, Japan; 2 Department of Parasitology, Institute of Tropical Medicine (NEKKEN), Nagasaki University, Nagasaki, Japan; 3 The Joint Usage/Research Center on Tropical Disease, Institute of Tropical Medicine (NEKKEN), Nagasaki University, Nagasaki, Japan; 4 Department of Immunology and Genomics, Osaka City University Graduate School of Medicine, Osaka, Japan; 5 Division of Innate Immune Regulation, International Research and Development Center for Mucosal Vaccines, The Institute of Medical Science, The University of Tokyo, Tokyo, Japan

**Keywords:** IL-33, *Schistosoma mansoni*, Worm maturation, Type 2 immunity, Egg-induced pathology

## Abstract

**Background:**

Schistosomes are trematode worms that dwell in their definitive host’s blood vessels, where females lay eggs that need to be discharged into the environment with host excreta to maintain their life-cycle. Both worms and eggs require type 2 immunity for their maturation and excretion, respectively. However, the immune molecules that orchestrate such immunity remain unclear. Interleukin (IL)-33 is one of the epithelium-derived cytokines that induce type 2 immunity in tissues. The aim of this study was to determine the role of IL-33 in the maturation, reproduction and excretion of *Schistosoma mansoni* eggs, and in the maintenance of egg-induced pathology in the intestines of mice.

**Methods:**

The morphology of *S. mansoni* worms and the number of eggs in intestinal tissues were studied at different time points post-infection in *S. mansoni*-infected IL-33-deficient (IL-33^−/−^) and wild-type (WT) mice. IL-5 and IL-13 production in the spleens and mesenteric lymph nodes were measured. Tissue histology was performed on the terminal ilea of both infected and non-infected mice.

**Results:**

Worms from IL-33^−/−^ and WT mice did not differ morphologically at 4 and 6 weeks post-infection (wpi). The number of eggs in intestinal tissues of IL-33^−/−^ and WT mice differed only slightly. At 6 wpi, IL-33^−/−^ mice presented impaired type 2 immunity in the intestines, characterized by a decreased production of IL-5 and IL-13 in mesenteric lymph nodes and fewer inflammatory infiltrates with fewer eosinophils in the ilea. There was no difference between IL-33^−/−^ and WT mice in the levels of IL-25 and thymic stromal lymphopoietin (TSLP) in intestinal tissues.

**Conclusions:**

Despite its ability to initiate type 2 immunity in tissues, IL-33 alone seems dispensable for *S. mansoni* maturation and its absence may not affect much the accumulation of eggs in intestinal tissues. The transient impairment of type 2 immunity observed in the intestines, but not spleens, highlights the importance of IL-33 over IL-25 and TSLP in initiating, but not maintaining, locally-induced type 2 immunity in intestinal tissues during schistosome infection. Further studies are needed to decipher the role of each of these molecules in schistosomiasis and clarify the possible interactions that might exist between them.
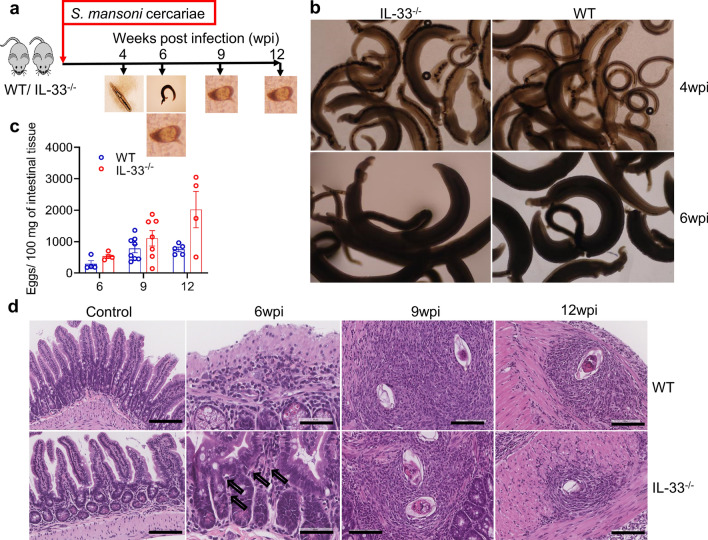

## Background

Schistosomes are blood-dwelling trematode worms that affect over 250 million people in the world, of whom 201.5 million live in sub-Saharan Africa [1]. Among the several schistosome species that exist, three, namely *Schistosoma haematobium*, *S. japonicum* and *S. mansoni*, are the main cause of schistosomiasis in humans [2]. The first of these causes urogenital schistosomiasis and the latter two cause hepato-splenic schistosomiasis [2]. *Schistosoma haematobium* worms live in perivesical vein plexuses, and *S. japonicum* and *S*. *mansoni* live in mesenteric veins, where females lay hundreds to thousands of eggs per day [3]. About half of these eggs are washed into the liver by blood flow from the mesenteric veins; of the remainder, one-third to one-half succeed in reaching the intestinal lumen to be discharged into the environment with the host’s feces, while the remaining eggs are trapped in intestinal tissues where they die, either killed by the host’s immune system or of natural death [4, 5].

Through their excretory–secretory products (ESP), such as the interleukin (IL)-4-inducing principle of *S. mansoni* eggs (IPSE/α1) [6, 7] and omega-1 (ω1) [8] from *S. mansoni* and their homologs from *S. haematobium* [9] and *S. japonicum* [10], tissue-trapped eggs elicit strong and vigorous type 2 cell-mediated immunity that induces perioval granuloma formation and leads to fibrosis [11, 12], pathological characteristics of a patent schistosome infection. While this immune response is thought to be beneficial for the host, especially in the liver where it may protect hepatic cells from toxic effects of egg-derived ESP, it also plays a major role in the development of liver pathology [13]. In contrast, in addition to being protective for and yet smiting the host with the pathology, granulomas in intestines play a beneficial role for the parasite, favoring the escape of eggs from the host through the intestinal wall [14, 15].

Eggs are not the sole inducers of type 2 immunity in schistosomiasis, as studies have reported type 2 immune responses during prepatent schistosomiasis infection before egg deposition by female worms begins [16, 17]. The type 2 immunity during the prepatent schistosome infection was later found to be essential for the maturation of the worms, as the injection of IL-4, the T-helper 2 (Th2) polarizing cytokine, in schistosome-infected recombination activating gene (RAG)-deficient mice, in which schistosome worms fail to mature and reproduce due to the lack of functional CD4^+^ T cells, restored the worm maturation process and egg deposition [18].

Emerging evidence indicates that the induction of type 2 immunity in tissues is not solely dependent on IL-4, with type 2 immunity shown also to be induced through the activation of group 2 innate lymphoid cells (ILC2) by the epithelium-derived cytokines IL-25, IL-33 and thymic stromal lymphopoietin (TSLP). Activated ILC2, in turn, produce abundant amounts of type 2 effector cytokines IL-4, IL-5, IL-9 and IL-13 [19–22] and, through the expression of the class II major histocompatibility complex (MHC II) [23] and OX40L [24], interact with CD4^+^ T cells to potentiate such type 2 immune responses. ILC2 were also found to initiate the adaptive type 2 immunity in an IL-4-independent manner by inducing IL-13-dependent activation and migration of dendritic cells to the draining lymph nodes where they polarize naïve CD4^+^ T cells into Th2 cells [25].

However, the role of ILC2-activating cytokines IL-25, IL-33 and TSLP in schistosomiasis remains less understood. Focusing on the liver pathogenesis during *S. japonicum* infection, two studies showed that IL-33 contributes to the development of pathology* via* the induction of type 2 immune responses in infected mice [26, 27]. Indeed, studies have shown that IL-33 plays a critical role in the development of liver pathology through the alternative activation of macrophages (M2) [27] and the activation of hepatic stellate cells by ILC2-derived IL-13 [28]. Moreover, Yu et al. [26] found that the injection of exogenous IL-33 into *S. japonicum*-infected mice led to an increased worm burden at the sixth week of infection without affecting their fecundity, suggesting that IL-33 might play a role in the migration and maturation of schistosome worms. Whether endogenous IL-33 plays a role in schistosome maturation and reproduction is not known.

As IL-33 is known to induce and/or amplify M2 polarization of macrophages [27, 29–31] which are essential for the excretion of schistosome eggs [14, 15], we considered the possibility that in addition to contributing to the maturation of schistosome worms through the induction of type 2 immunity during prepatent schistosome infection, IL-33 may also play a role in the accumulation of *S. mansoni* eggs in the intestinal tissues. We hypothesized that IL-33 deficiency would impair the maturation of *S. mansoni* worms and possibly also lead to the accumulation of more eggs in the intestinal tissues [14, 32, 33], and that type 2 immunity would be impaired in the absence of IL-33. In the study reported here, we show that IL-33 is in fact dispensable for the maturation of *S. mansoni* worms and that its absence does not affect much the number of eggs accumulated in the intestinal tissues. Also, our findings support the notion that IL-33 might be most potent in initiating, but not maintaining, type 2 immunity in tissues and that to maintain type 2 immunity once initiated, IL-33 may need the synergy of IL-25 and TSLP and/or of CD4^+^ Th2-derived effector cytokines.

## Methods

### Parasite, mice and infection

A Puerto Rican strain of *S. mansoni* was maintained in the laboratory by passage between *Biomphalaria glabrata* snails and ICR mice. BALB/cCrSlc (hereinafter referred to as BALB/c) mice were purchased from Japan SLC (Hamamatsu, Shizuoka, Japan) and maintained in specific pathogen-free conditions at Nagasaki University animal facilities. Provided by Professor Satoshi Uematsu (Osaka City University Graduate School of Medicine, Osaka, Japan), IL-33^−/−^ mice on the BALB/c background were bred and maintained in the same conditions as for WT BALB/c mice at Nagasaki University animal facilities. All mice were provided with water and food* ad libitum*. Female mice aged 8–12 weeks were subcutaneously infected [34] with 50 and 35 freshly shed *S. mansoni* cercariae for 9 and 12 weeks, respectively. Mice were sacrificed every 3 weeks from week 6 post-infection (6 wpi) onwards, except for worm morphology assessment where mice were also sacrificed at 4 wpi. To assess the production of IL-25, IL-33 and TSLP in intestinal tissues during *S. mansoni* infection, WT BALB/c mice were infected with *S. mansoni* cercariae as described above and sacrificed weekly from week 0 to week 4 post-infection, then every 2 weeks to 12 wpi.

### Worm morphology and number

Adult *S. mansoni* worms were obtained by portal vein perfusion and fixed with 4% neutral buffered formalin (NBF) [35], following which their morphology was assessed under an inverted light microscope at 40× magnification and their number counted. Briefly, the portal vein was cut at its base under the liver, then the left cardiac ventricle was perfused with 30 mL of saline citrate (7.5 g of sodium citrate and 8.5 g of sodium chloride in milliQ) [34], followed by perfusion with 30 mL of phosphate buffered saline (PBS). The mesenteric veins were thoroughly checked for manual retrieval of worms that failed to wash out during perfusion.

### Tissue eggs and eggs per worm pair numbers

Livers and intestines were harvested and digested with 4% KOH at 37 °C for 14 h. Briefly, livers were weighed and digested with 10 mL of 4% KOH, and intestines were cleansed of fecal matters, opened longitudinally, thoroughly washed with PBS, weighed and then digested as described for livers [34]. After digestion, samples were centrifuged for 5 min at 2000 rpm and room temperature. Eggs were counted in 50 µL of thoroughly mixed pellet suspension under the light microscope at 40× magnification and related to the organ weight. The number of eggs per worm pair was obtained by dividing the total number of tissue eggs per mouse by the number of worm pairs from the same mouse.

### Egg isolation and production of *S. mansoni* soluble egg antigen

Eggs were isolated from the livers of *S. mansoni*-infected ICR mice and frozen at − 30 °C until use. Briefly, 8 weeks after infection with 200 *S. mansoni* cercariae, livers of the infected mice were removed after the portal perfusion, washed with PBS, minced with sterile scissors and then homogenized in 1× PBS using the IKA T25 digital Ultra Turrax homogenizer (IKA-Werke GmbH & Co., Staufen, Germany). The homogenates were centrifuged for 5 min at 1500 rpm and room temperature, digested twice at 37 °C with shaking at 120 rpm, first with 1 mg/mL of Actinase E (Funakoshi, Tokyo, Japan) for 3 h, then with 0.1 mg/mL of Actinase E (Funakoshi) and 0.5 mg/mL of collagenase (Wako Pure Chemicals, Osaka, Japan) for 2 h. Obtained egg suspensions were filtered through a series of sieves (425, 180, 106 and 45 µm) [34]. The eggs retained on the smallest sieve (45 µm) were washed onto a Petri dish [29]. By swirling the dish, mature eggs were concentrated in the center of the dish and collected with a wide-bore pipette tip [34]. After settling, the supernatant was discarded and eggs were dry frozen at − 30 °C until use.

For the production *S. mansoni* soluble egg antigen (*Sm*SEA), frozen eggs were thawed on ice, resuspended in ice-cold PBS containing 10 µg/mL of leupeptin, then homogenized on ice using a handheld sterile glass Teflon homogenizer. The homogenate was subjected to five freeze (− 80°C) and thaw (on ice) cycles, incubated at 4 °C overnight with rotation, then centrifuged for 1 h at 30,000 *g* and 4 °C. The supernatant was collected in new tubes on ice, dialyzed in PBS three times at 4 °C for 2 and 4 h and overnight, respectively, by using a Slide-A-Lyzer Dialysis Cassette (Thermo Fisher Scientific, Rockford IL, USA) as per the manufacturer’s instructions. The protein concentration was determined by the Bicinchoninic acid (BCA) method (Pierce BCA Protein Assay, Thermo Fisher Scientific). The solution was filter-sterilized through a 0.2-µm filter, aliquoted and stored at − 30 °C until use.

### Cell stimulation and cytokine measurement

Immune cells were isolated from spleens and mesenteric lymph nodes (MLNs) of infected and non-infected wild-type (WT) and IL-33^−/−^ mice and stimulated with *Sm*SEA. Briefly, spleens were crushed and filtered through a 70-µm-mesh cell strainer, washed with Hank’s balanced salt solution (HBSS), then resuspended in complete RPMI medium (containing 10% fetal bovine serum, 100 units/mL of penicillin and 100 µg/mL of streptomycin, 55 µM of 2-mercaptoethanol, HEPES [4-(2-Hydroxyethyl)-1-piperazineethanesulfonic acid] and l-glutamine). MLNs were then filtered through a 40-µm-mesh cell strainer and processed as above. The cells (1.0 × 10^6^ cells per well) were plated in a flat bottomed 96-well plate, stimulated with 50 µg/mL of *Sm*SEA in a 5% CO_2_ incubator at 37 °C for 72 h. The plates were then stored at − 30 °C until use. The concentrations of IL-5 and IL-13 were measured in the culture supernatants by an enzyme-linked immunosorbent assay (ELISA) as per the manufacturer’s instructions (DuoSet ELISA; R&D Systems, Minneapolis, MN, USA).

### Tissue cytokines

Small intestines were harvested in ice-cold PBS. After fecal matter had been removed, the intestines were opened along their axis, thoroughly washed with ice-cold PBS, cut into small pieces and homogenized in 3 mL of ice-cold HBSS (containing 10 µg/mL of leupeptin and 0.1 mM/mL of phenylmethylsulfonyl fluoride) with the gentleMACS Octo Dissociator (Miltenyi Biotec GmbH, Bergisch Gladbach, Germany) using the Protein_01 program. The homogenates were then centrifuged for 20 min at 20,000 *g* and 0 °C [36], and the supernatants were collected, aliquoted and stored at − 30 °C until use. The concentrations of IL-25, IL-33 and TSLP were measured in the supernatants by an ELISA according to the manufacturer’s instructions (DuoSet ELISA; R&D Systems, Inc.).

### Histology

A 1-cm-long fragment of terminal ileum was cut from each mouse, close to the secum of both infected and non-infected mice, cleaned of fecal matter and fixed in 10% NBF until use. Samples were sent to the Division of Cell Function Research Support, Biomedical Research Support Center at Nagasaki University School of Medicine, for tissue processing. Slide-embedded hematoxylin and eosin-stained tissue sections were scanned at 40× magnification using Aperio CS2 Scanner (Leica Biosystems Imaging, Leica Biosystems, CA, USA), and digital images were analyzed using Aperio ImageScope version 12.4.3 software (Leica Biosystems Imaging, Leica Biosystems). The number and size of granuloma areas were counted and measured, respectively. The intestinal wall thickness was measured at three different places. The abundance of eosinophils in the inflammatory infiltrates was visually determined. All of the measurement results were compared between IL-33^−/−^ and WT.

### Statistical analysis

Data normality was determined by the Shapiro–Wilk test. Using GraphPad Prism version 8.4.2 for Windows (GraphPad Software, San Diego, CA, USA), we performed Welch’s t-test or the Mann-Whitney U-test to compare IL-33^−/−^ and WT mice. Statistical significance was set at *P* < 0.05. Unless otherwise stated, all data are presented as mean with standard error of the mean and are representative of at least two independent experiments with similar results.

## Results

### IL-33 deficiency does not affect *S. mansoni* worm maturation and the number of eggs in intestinal tissues

**Fig. 1:**
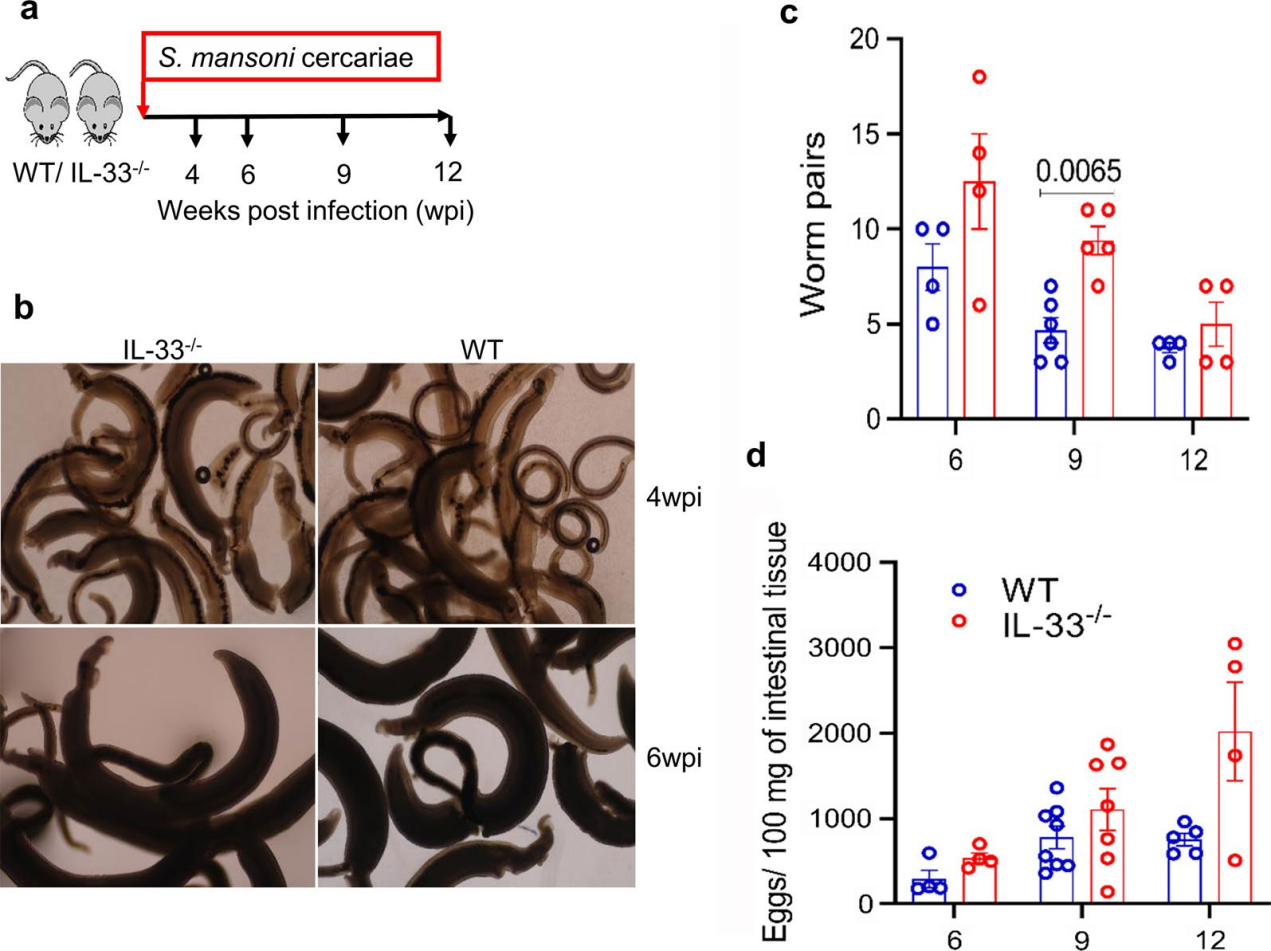
Interleukin-33 (*IL-33*) deficiency does not affect *Schistosoma mansoni* worm maturation and the number of eggs in intestinal tissues. **a** Female IL-33^−/−^ and wild-type (*WT*) BALB/cCrSlc (*BALB/c*) mice (4–8 animals per group) were subcutaneously infected with 50 and 35 *S. mansoni* cercariae for 9 and 12 weeks, respectively, and sacrificed at 4, 6, 9 and 12 weeks post-infection (*wpi*) to assess the morphology of worms and count the number of worm pairs and tissue eggs. **b** Representative photographs of the morphology of worms recovered from both mouse genotypes at weeks 4 (upper panels) and 6 (lower panels) at ×40 magnification. **c** Number of worm pairs from both IL-33^−/−^ and WT mice. **d** Number of eggs in intestinal tissue. Groups were compared using the unpaired two-tailed t-test with Welch’s correction. Experiments were replicated at least three times. Data are representative of 2 independent experiments with similar results and are presented as the mean with standard error of the mean (SEM). Significance (*P* value) is indicated above connector bars between appropriate groups. Mouse groups were compared using the Mann–Whitney test at the *P* < 0.05 level of significance

Schistosome worms are characterized by their dependence on the host immune system, particularly type 2 immunity, for their maturation, reproduction and egg excretion [15, 18, 35, 37], indicating the importance of type 2 immunity in the biology of schistosomes. Because IL-33 is known to induce type 2 immunity independently of IL-4 [38] and in light of the results of a recent study [26] which reported increased *S. japonicum* worm numbers after injection of exogenous IL-33 into infected mice, we decided to investigate whether IL-33 deficiency would compromise the maturation of *S. mansoni* worms by comparing the morphology and number of worm pairs between IL-33^−/−^ and WT mice. We therefore infected IL-33^−/−^ and WT BALB/c mice with *S. mansoni* cercariae and sacrificed them at indicated post-infection time points (Fig. 1a). As shown in Fig. 1b, in terms of morphology, there was no difference between worms recovered from IL-33^−/−^ and those recovered from WT mice. Although the number of worm pairs tended to be higher in IL-33^−/−^ mice during the course of infection, this difference was statistically significant only at the ninth week of infection (*U* = 0.5, *P* = 0.0065; Fig. 1c). The number of eggs per worm pair, calculated by dividing the raw number of worm pairs by the raw number of tissue (liver and intestinal) eggs, seemed to be comparable between IL-33^−/−^ and WT mice during the whole course of infection (*t*_(5)_ = 0.184, *P* = 0.861; *t*_(9)_ = 1.972, *P* = 0.081; *t*_(3)_ = 0.919, *P* = 0.423, at 6, 9 and 12 wpi, respectively; Additional file 1: Figure S1a).

To evaluate whether IL-33 deficiency may affect the accumulation of eggs in the intestinal tissues, we compared the number of eggs in intestinal tissues between IL-33^−/−^ and WT mice every 3 weeks from week 6 to week 12 post infection. Similar to the observations on the number of worm pairs, the number of eggs in intestinal tissues tended to be higher in IL-33^−/−^ mice than in WT mice (Fig. 1d), but no statistical difference was found between both mouse genotypes (*U* = 3, *P* = 0.2; *U* = 18, *P* = 0.281; *U* = 5, *P* = 0.285 at 6, 9 and 12 wpi, respectively). Because several previous studies had reported a pathogenic role for IL-33 in egg-induced liver pathology by an increased number of liver tissue eggs [26, 27], we investigated whether IL-33 deficiency would affect the number of eggs in liver tissues of IL-33^−/−^ compared to WT mice. We found that although IL-33^−/−^ mice tended to have higher number of eggs than WT mice (Additional file 1: Figure S1b), there was no statistical difference in liver egg numbers between both mouse genotypes (*t*_(4)_ = 2.097, *P* = 0.097; *t*_(9)_ = 0.397, *P* = 0.7; *t*_(4)_ = 1.298, *P* = 0. 2653 at 6, 9 and 12 wpi, respectively). Taken together, these data indicate that IL-33 is dispensable for the maturation of *S. mansoni* worms and that its absence may have a negligible effect on the accumulation of eggs in the intestinal tissues.

### IL-33 deficiency is associated with transitory impairment of type 2 immunity in mesenteric lymph nodes of *S. mansoni*-infected mice

**Fig. 2:**
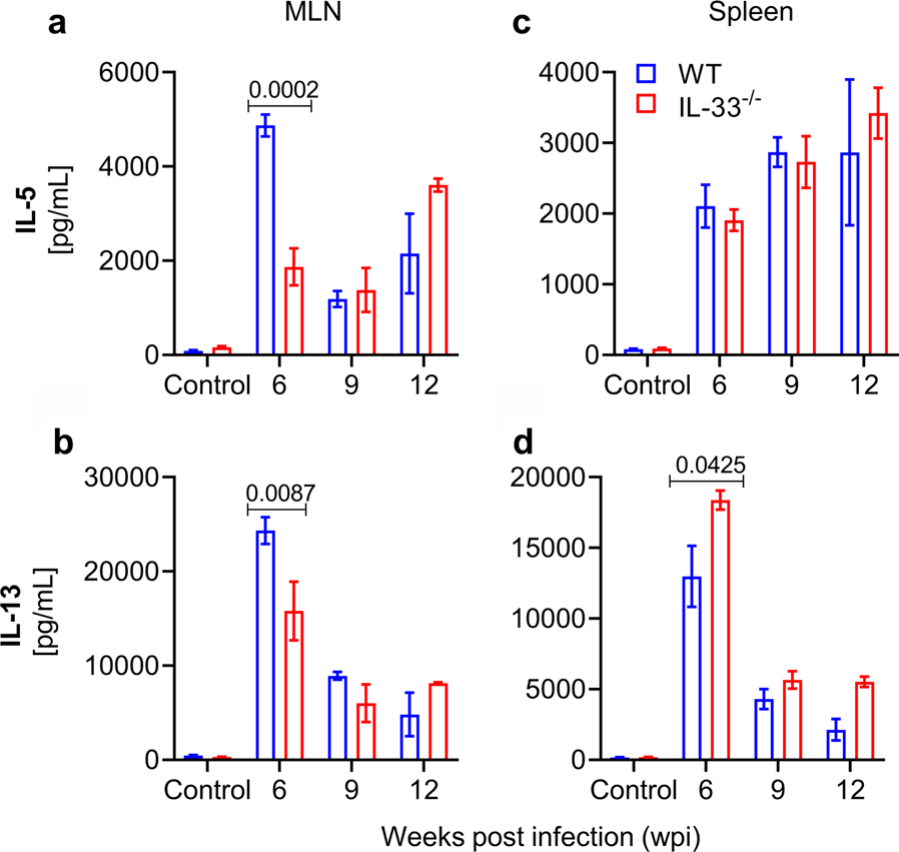
IL-33 deficiency is associated with transitory impairment of type 2 immunity in mesenteric lymph nodes (*MLN*) of infected mice. Female IL-33^−/−^ and WT BALB/c mice (4–8 animals per group) were subcutaneously infected with 50 and 35 *S. mansoni* cercariae for 9 and 12 weeks, respectively, and sacrificed at 6, 9 and 12 wpi. Immune cells were isolated from MLNs and spleens of infected and non-infected (control) mice and stimulated with *S. mansoni* soluble egg antigens for 72 h at 37 °C, 5% CO_2_. IL-5 and IL-13 cytokines were measured in cell culture supernatants by enzyme-linked immunosorbent assay (ELISA). **a** IL-5 from MLNs. Comparison by unpaired two-tailed t-test with Welch’s correction for 6 wpi. **b** IL-13 from MLNs. **c** IL-5 from spleens. **d** IL-13 from spleens. Comparison by unpaired two-tailed t-test with Welch’s correction for 6 wpi. Data are representative of two independent experiments with similar results and are presented as the mean with SEM. Significance (*P* value) is indicated above connector bars between appropriate groups. Mouse groups were compared using the Mann-Whitney test at the *P* < 0.05 level of significance

Compared with IL-25 and TSLP, IL-33 is known to be the stronger molecule for inducing type 2 immunity through the activation of ILC2 and macrophages [39, 40]. Therefore, we assessed whether IL-33 deficiency would impair type 2 immunity in intestines. We isolated immune cells from MLNs of *S. mansoni*-infected IL-33^−/−^ and WT mice, stimulated them with *Sm*SEA for 72 h and then measured IL-5 and IL-13 cytokines by ELISA. As expected, IL-33 deficiency impaired the production of IL-5 and IL-13 in the MLNs of infected mice in response to stimulation with *Sm*SEA at 6 weeks of infection (*t*_(8)_ = 6.595, *P* = 0.0002 for IL-5 and *U* = 2, *P* = 0.008 for IL-13; Fig. 2a, b). However, this impairment was not sustained during the course of infection as it disappeared at subsequent infection time points (*U* = 6, *P* = 0.6857; *U* = 1, *P* = 0.4 for IL-5 and *U* = 3, *P* = 0.2; *U* = 1, *P* = 0.4 for IL-13 at 9 and 12 wpi, respectively; Fig. 2a, b). To verify whether this impairment was limited to the intestines or was systemic, we isolated immune cells from spleens of infected mice and measured IL-5 and IL-13 in the supernatants after 72 h of stimulation with *Sm*SEA. Surprisingly, while there was no statistical difference between IL-33^−/−^ mice and WT mice in terms of the production of IL-5 in spleens during the course of infection (*U* = 19, *P* = 0.1949; *U* = 8, *P* > 0.999; *U* = 2, *P* = 0.8 at 6, 9 and 12 wpi, respectively; Fig. 2c), there was a statistically significant difference in the production of IL-13 at the sixth week of infection (*t*_(8)_ = 2.39, *P* = 0.042; Fig. 2d).

### IL-33 deficiency transiently attenuated egg-induced pathology in intestines of *S. mansoni*-infected mice

**Fig. 3:**
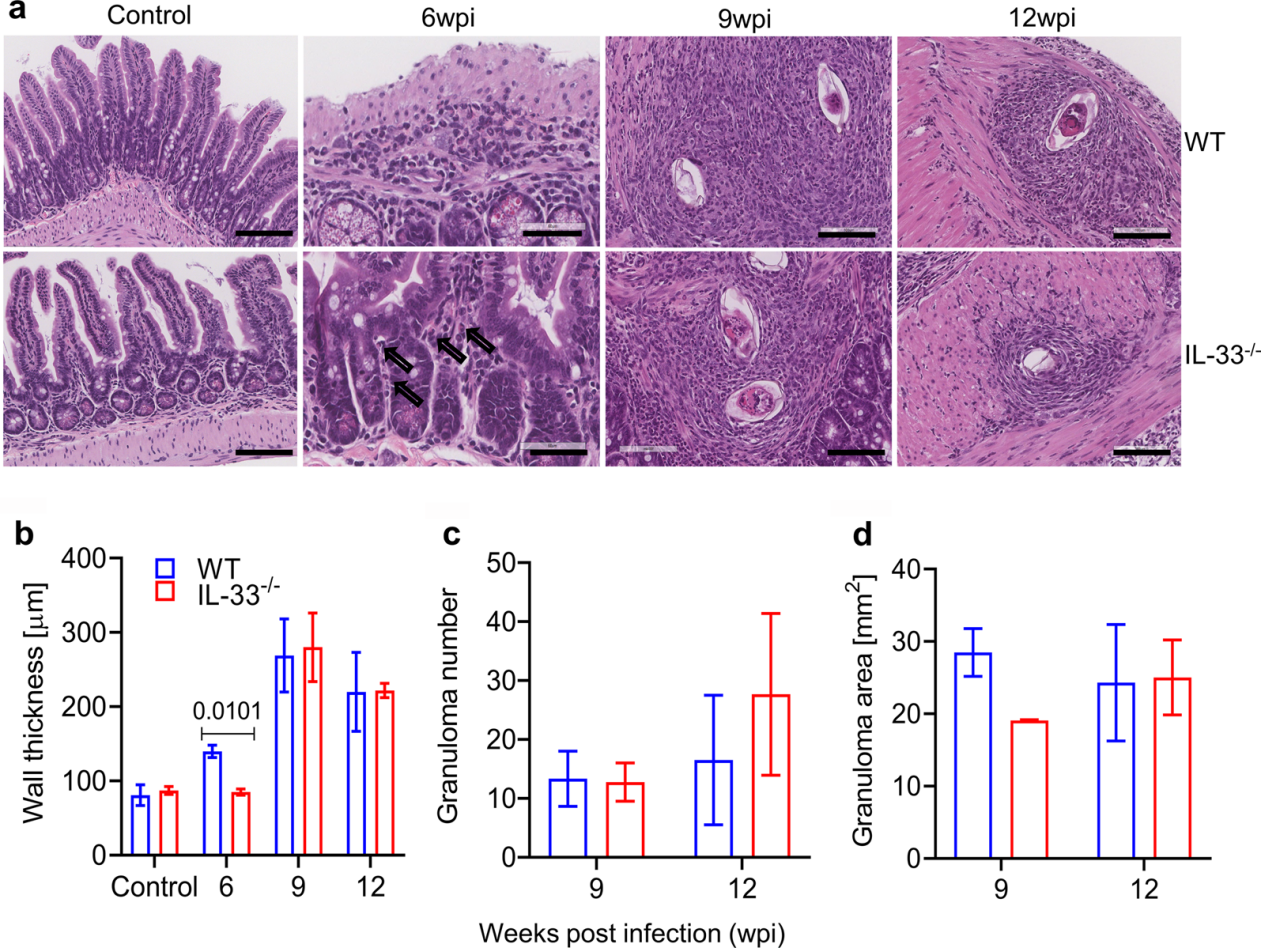
IL-33 deficiency transiently attenuated egg-induced pathology in the intestines of infected mice. Female IL-33^−/−^ and WT BALB/c mice were subcutaneously infected with 50 and 35 *S. mansoni* cercariae for 9 and 12 weeks, respectively, and sacrificed at 6, 9 and 12 wpi. Terminal ileum fragment was obtained from each infected and non-infected mouse (3 animals per group), fixed in 10% neutral buffered formalin, and processed for histology. **a** Representative hematoxylin and eosin-stained histological sections of terminal ileum from infected and non-infected WT (upper panels) and IL-33^−/−^ (lower panels) mice. Scale bar: 200 µm for controls, 60 µm for 6 wpi 100 µm for 9- and 12 wpi. Arrows indicate eosinophils in the inflammatory inflitrate. **b** Intestinal wall thickness (mucosal layer excluded) in microns. Unpaired two-tailed t-test with Welch’s correction was used to compare mouse genotypes in controls and infected mouse groups at 6 and 9 wpi, and the Mann–Whitney test was used to compare infected mouse groups at 12 wpi. **c** Number of granulomas per tissue section. **d** Granuloma area (mm^2^). Data are presented as the mean with SEM. Significance (*P* value) is indicated above connector bars between appropriate groups. The level of significance was set at *P * < 0.05

Although schistosome worms also induce type 2 immunity [16, 17], eggs remain the most potent inducers of type 2 immunity and the main cause of the pathology in the liver and intestines of infected definitive hosts [11–13]. A pathogenic role for IL-33 in liver pathology during schistosome infections has been reported [26–28]. Although none of these studies reported on the role of IL-33 in the development of intestinal pathology, studies related to inflammatory bowel diseases have found controversial roles for IL-33 in the development and/or exacerbation of these diseases, with some reporting a protective role for IL-33 [41] and others incriminating it in the development or exacerbation of these diseases [42, 43]. Given this uncertainty, we investigated whether IL-33 deficiency would compromise the development of egg-induced pathology in intestinal tissues of infected mice. As shown in Fig. 3a, b, IL-33 deficiency was transiently associated with attenuated type 2 inflammatory responses in the terminal ilea of IL-33^−/−^ mice compared to WT mice, characterized by less infiltration of intestinal tissues by inflammatory cells and a wall thickness similar to that of non-infected mice at the sixth week of infection (*t*_(3)_ = 5.897, *P* = 0.010; Fig. 3b). Moreover, the inflammatory infiltrates in IL-33^−/−^ mice contained fewer eosinophils than those in WT mice at the sixth week of infection (Fig. 3a). Both mouse genotypes did not differ in terms of granuloma number (*U* = 3, *P* > 0.999 and *U* = 2, *P* = 0.8 at 9 and 12 wpi, respectively; Fig. 3c) and area (*U* = 0, *P* = 0.2 and *U* = 3, *P* > 0.9999 at 9 and 12 wpi, respectively; Fig. 3d). Taken together, these data suggest that IL-33 may be more important in initiating—but not maintaining−type 2 immunity at mucosal barriers than needed systemically and that it is not needed for the maintenance of schistosome egg-induced pathology in intestines. These results prompted us to speculate that IL-25 and TSLP expression might be upregulated in this setting to compensate for the absence of IL-33 at later infection time points in intestines.

### There is no change in IL-25 and TSLP production in the absence of IL-33 in intestines of infected mice

**Fig. 4:**
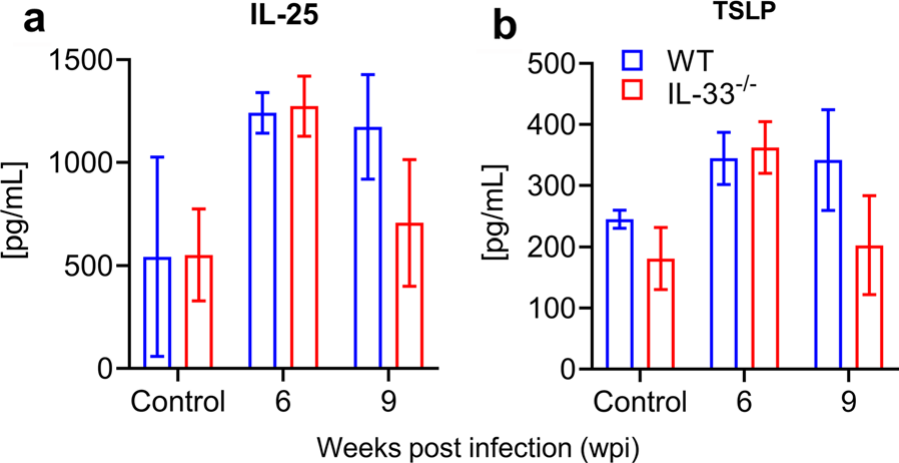
There is no change in IL-25 and thymic stromal lymphopoietin (*TSLP*) production in the intestines of infected mice. Female IL-33^−/−^ and WT BALB/c mice (3 animals per group) were subcutaneously infected with 50 *S. mansoni* cercariae for 9 weeks and sacrificed at 6 and 9 wpi. Small intestines were homogenized with the gentleMACS Octo Dissociator and the cytokines were measured in the homogenate supernatants by ELISA. **a** IL-25 from infected and non-infected mice. **b** TSLP from infected and non-infected mice. Experiments were replicated twice with similar results. Data are presented as the mean with SEM. Mouse genotypes were compared using the Mann–Whitney test for non-infected mice and the unpaired two-tailed t-test with Welch’s correction for infected mice

Individually or synergistically, IL-25, IL-33 and TSLP are known to induce tissue type 2 immune responses in different homeostatic and pathologic conditions [39, 40, 44, 45]. The existence of possible interactions between these cytokines has also been suggested [40, 45]. We therefore reasoned that, due to IL-33 deficiency, there might be compensatory changes in IL-25 and/or TSLP production in *S. mansoni*-infected IL-33^−/−^ mice compared to WT mice. As shown in Fig. 4, in the small intestinal tissues there was no statistically significant difference in the levels of IL-25 (*U* = 3, *P* > 0.999; *t*_(4)_ = 0.185, *P* = 0.863; *t*_(4)_ = 1.169, *P* = 0.309 for non-infected and infected mice at 6 and 9 wpi, respectively; Fig. 4a) and TSLP (*U* = 1, *P* = 0.666; *t*_(4)_ = 0.296, *P* = 0.781; *t*_(4)_ = 1.205, *P* = 0.294 for non-infected and infected mice at 6 and 9 wpi, respectively; Fig. 4b) between IL-33^−/−^ and WT mice, indicating that there is no compensatory changes in IL-25 and TSLP production in the absence of IL-33. Although the levels of IL-25 and TSLP expression in intestinal tissue homogenates tended to increase with *S. mansoni* infection compared to non-infected mice, infected mice did not produce enough of these cytokines to reach a statistically significant difference (Fig. 4a, b).

Previous studies reported an increase in IL-33 levels in the sera of individuals with *S. japonicum* infection compared to non-infected individuals [27]. In mice, this increase, which starts around week 4 of infection, reaches its peak around 8 wpi [26], corresponding with the oviposition period. This timeline may indicate that schistosome eggs are the major inducers of IL-33 release in schistosome infection settings. However, due to the functional redundancy of IL-33 with respect to IL-25 and TSLP [46], we investigated the kinetics of production of these cytokines during an *S. mansoni* infection. Thus, we infected only WT BALB/c mice with *S. mansoni* cercariae and checked for the release of IL-25, IL-33 and TSLP in their intestinal tissues. While the levels of IL-33 remained constantly higher even in naïve mice, levels of IL-25 and TSLP tended to increase with oviposition (Additional file 2: Figure S2), indicating that *S. mansoni* eggs may induce the release of IL-25 and TSLP, but not of IL-33, in the intestines of infected mice.

## Discussion

Studies have shown that both schistosome worms and eggs induce type 2 immunity, which is essential for their maturation, reproduction and egg excretion [14–18]. Deficiency in CD4^+^ Th2 cells and their effector cytokines IL-4 and IL-13 has been shown to substantially decrease or completely abrogate egg excretion as a consequence of impaired worm maturation or failed signaling by type 2 effector cytokines [15, 47–49]. Moreover, accumulating evidence suggests that ILC2 and their activating cytokines IL-25, IL-33 and TSLP induce adaptive type 2 immunity independently of IL-4 [23–25]. While no data are as yet available on the induction of IL-25, IL-33 and TSLP release by migrating schistosomula, it has been reported that schistosome eggs induce and/or enhance the production of IL-25 and IL-33 [50, 51], indicating that schistosome eggs may be orchestrating their excretion by inducing the production of alarmin cytokines IL-25, IL-33 and TSLP to trigger type 2 immunity, which is essential to their excretion. In the present study we found that IL-33 deficiency did not affect the maturation of worms as at both the early (4 wpi) and later (6 wpi) time points the morphology did not differ between worms recovered from IL-33^−/−^ and those recovered from WT mice (Fig. 1b), suggesting that IL-33 may not be required for schistosome worm maturation. Consequently, despite a higher number of worm pairs in IL-33^−/−^ mice during the course of infection, with a statistical difference at the ninth week of infection (Fig. 1c), the number of eggs per worm pair did not differ between the mouse genotypes (Additional file 1: Figure S1a). Also, despite the number of tissue eggs being higher in IL-33^−/−^ mice than in WT mice, the difference was not statistically significant between both mouse genotypes (Fig. 1e; Additional file 1: Figure S1b). The number of eggs in tissues paralleled the number of worm pairs in both mouse genotypes, explaining the similarity in the number of eggs per worm pairs despite a higher number of worm pairs and tissue egg numbers in IL-33^−/−^ mice.

Lungs have been shown to be the major site of attrition for migrating schistosomula, achieved through different mechanisms, including the penetration of schistosomula into alveoli and their expulsion through the pulmonary tract, and immune-mediated killing of schistosomula [52, 53]. Lungs are also one of the tissues where constitutive IL-33 is abundantly expressed in mice [54]. Schistosomula that enter the alveoli may damage the bronchoalveolar epithelium, inducing the release of IL-33 which, in turn, may initiate an anti-schistosomula immune response, thus contributing to the attrition of schistosomula. This process can result in IL-33^−/−^ mice appearing to be more permissive or susceptible to *S. mansoni* infection, explaining the apparent lower number of worm pairs in WT mice compared to IL-33^−/−^ mice (Fig. 1c).

We did not determine worm length, the proportion of single worms (males or females), the proportion of females in pairs [18, 55] or the number of eggs in the feces [14, 15]. However, based on the known morphology of worms [35, 56] and known intestinal tissue egg numbers [14, 32, 33], we believe that our study design is an appropriate alternative approach to looking at the effect of IL-33, as a potent initiator of type 2 immunity necessary for schistosome worm maturation, on the maturation of *S. mansoni* worms and the accumulation of eggs in intestinal tissues in the case of failed expulsion [14, 32, 33].

Yu et al. [26] reported that the injection of *S. japonicum*-infected mice with exogenous IL-33 increased the number of worms recovered at the sixth week of infection and also exacerbated the liver pathology by increasing the number and size of liver granulomas. This finding may simply mean that as endogenous IL-33 plays a role in the development of egg-induced liver pathology [26–28], injecting exogenous IL-33 would exacerbate its pathogenic effects. In the present study, we did not find any statistically significant difference in the number of eggs per worm pair between IL-33^−/−^ and WT mice (Additional file 1: Figure S1a). These results corroborate those reported by Yu et al. [26] as they did not find a difference in the number of eggs per female worm. This result indicates that IL-33 alone may play a negligible role in worm maturation and the expulsion of eggs.

While studies related to inflammatory bowel diseases have reported controversial roles for IL-33 in the development and/or exacerbation of these diseases, with some reporting a protective role for IL-33 [31, 41] and others incriminating it in the development or exacerbation of these diseases [42, 43], to the best of our knowledge there has been no report on the role of this cytokine in intestinal pathology during schistosomiasis. Although IL-33 seemed dispensable for *S. mansoni* worm maturation and the excretion of their eggs, we sought to determine whether it may play a significant role in the development of egg-induced pathology in the intestines of infected mice, as it does in the liver [26–28]. We found that the absence of IL-33 transiently impaired type 2 immunity in the small intestines of IL-33^−/−^ mice, but not in their spleens, characterized by impaired production of IL-5 and IL-13 cytokines in MLNs in response to stimulation with *Sm*SEA (Fig. 2) and attenuated egg-induced inflammation in the ilea of IL-33^−/−^ mice at 6 wpi (Fig. 3a, b). These results are in line with findings by Vannella et al. [46] who, focusing on the role of alarmin cytokines IL-25, IL-33 and TSLP in the development and maintenance of type 2 cytokine-driven inflammation and fibrosis in lungs and liver, found that ablation of these cytokines singly had no significant ameliorating effect on liver pathology. However, when all three cytokines were ablated, a significant improvement of the pathology could be observed in the early phase of the infection, indicating functional redundancy among these cytokines. The trends observed in the present study are in the same direction, with the absence of IL-33 not affecting the development of pathology (Fig. 3a) nor the number and size of granulomas in the intestines of IL-33^−/−^ mice at time points beyond the 6 wpi (Fig. 3c, d). However, the difference between the study by Vannella et al. [46] and our study is that we started our observations at 6 wpi, when egg-induced type 2 immunity is still at its start, while Vannella et al. started their observations at 9 wpi when egg-induced type 2 immunity has already reached its peak. We believe that Vannella et al. [46] may have found a significant difference between IL-33 deficient mice and WT at an earlier stage of the infection, as observed in our study.

Despite IL-33 sharing functional redundancy with IL-25 and TSLP [46], the former remains the most potent of all three cytokines in inducing type 2 immunity [39, 40, 57]. In addition to inducing type 2 immunity by itself, IL-33 can also potentiate the type 2 immunity induced by IL-25 and TSLP [58]. Of all the cells that respond to IL-33, ILC2 and Th2 are the most important as through their production of abundant amounts of the type 2 cytokines IL-4, IL-5 and IL-13, they play the most important role in cell-mediated effector type 2 immunity [59, 60], characterized by, among others, the accumulation of M2 macrophages and eosinophils in affected tissues. Although dispersed in all tissues, ILC2 are more abundant in the lungs and intestinal tissues [61], where they are the first to be activated by IL-33, subsequently migrating to local draining lymph nodes to initiate adaptive type 2 immunity [25, 62]. Thus, it is understandable that the absence of IL-33 in IL-33^−/−^ mice at the early stage of the patent infection might have left ILC2 inactivated, leading to impaired type 2 immunity [59], as seen in the present study (Fig. 2a, b). In addition to acting through ILC2 and Th2 cells, IL-33 also acts directly on eosinophils, inducing their activation and expansion [63, 64]. Therefore, its absence in IL-33^−/−^ mice can explain the small number of eosinophils in the inflammatory infiltrates at 6 wpi (Fig. 3a, b) [65]. However, due to the persistence of egg-derived ESP [6–10] as eggs keep accumulating in the tissues, and to the fact that IL-25 and TSLP can induce type 2 immunity independently of IL-33 [51, 66–68], alternative mechanisms leading to the activation of both innate and adaptive type 2 immunity, including the taking over of ILC2 activation by IL-25 and TSLP and Th2-dependent effector pathways, might have been activated to compensate the absence of IL-33 (Figs. 2, 3). Together, these alternative mechanisms may have led to improved type 2 immunity at time points beyond 6 wpi.

Results from various studies have pointed to the existence of possible interactions between IL-25, IL-33 and TSLP [40, 45]. In one study, anti-IL-33 treatment and TSLP receptor deficiency blocked the infection-induced expression of IL-25 in lung epithelial cells, and* ex vivo* treatment of ILC2 with TSLP increased their expression of IL-25 and IL-33 receptors [45]. In another study, the authors noted that IL-25 shared with IL-33 many activities on macrophages without having additive effects, pointing toward the possible existence of common downstream signaling pathways for their biological activities [40]. This led us to postulate that IL-33 deficiency might be associated with a modified production of IL-25 and TSLP in the intestines of *S. mansoni*-infected IL-33^−/−^ mice. However, our results show no modification of intestinal production of IL-25 and TSLP as their levels in intestinal tissue homogenates did not differ between mouse genotypes (Fig. 4a, b), meaning that although they can, individually or synergistically, induce type 2 immunity, the absence of one may not affect the others in the schistosome infection settings or intestines. The nature of the interactions and conditions of their occurrence between IL-25, IL-33 and TSLP pointed out in the above-mentioned studies [40, 45] remain to be clarified.

Studies in humans and mice have reported an increase of IL-33 levels in the sera of individuals and animals infected with *S. japonicum* [27]. Also, these increased levels of IL-33 in serum were found to peak around the 8th week of infection in mice [26], corresponding to the peak of egg-induced immune responses, suggesting that through their ESP, eggs may be the main inducers of IL-33 release in schistosome infections. Indeed, Hams et al. [50, 51] reported that injection of *S. mansoni* eggs or the recombinant form of their derived components, namely ω1, induced the production of IL-25 and IL-33 in the lungs and fat tissue, respectively. Whether eggs in intestinal tissues induce the production of IL-33, IL-25 and TSLP is not known. We measured the levels of these alarmin cytokines in the intestinal tissue homogenates during *S. mansoni* infection in WT BALB/c mice and found that IL-33 levels remained constantly higher, even in non-infected mice (Additional file 2: Figure S2). In contrast, IL-25 and TSLP levels fluctuated over the course of infection, peaking around the tenth week of infection, with TSLP at much lower levels than IL-25 (Additional file 2: Figure S2). The start of an increase in levels of IL-25 and TSLP tended to correspond to that of oviposition, suggesting that the latter might be inducing the release of IL-25 and TSLP, but not of IL-33. Flamar et al. [62] recently reported that IL-33 expression was high in the small intestines of naïve mice, corroborating our findings, and indicating that IL-33 is constantly expressed in high amounts in mouse intestinal tissues.

## Conclusions

To the best of our knowledge, this is the first study to look at the role of IL-33 in the maturation of *S. mansoni* worms, as well as at the effect of its absence on the accumulation of eggs in the intestinal tissues. It is also the first study to report on the role that IL-33 may play in the maintenance of egg-induced type 2 immunity in intestines of *S. mansoni*-infected mice. The results show that IL-33 is dispensable for the maturation of *S. mansoni* and that its absence may have a negligible effect on the number of eggs accumulating in intestinal tissues when they fail to exit the intestines. Furthermore, due to transient impairment of type 2 immunity observed in the intestines but not spleens, this study highlights the importance of IL-33 over IL-25 and TSLP in initiating, but not maintaining, locally induced type 2 immunity in intestinal tissues in schistosome infections. These results corroborate previously reported findings that IL-25, IL-33 and TSLP may be sharing a partial functional redundancy in their ability to maintain tissue-induced type 2 immunity. Their combined or sequential ablation might be the best option to decipher the role of each of them in schistosomiasis and clarify the possible interactions that might exist between them.

## Supplementary Information

**Additional file 1: Figure S1.** IL-33 deficiency does not affect the number of eggs produced per *S. mansoni* worm pair and the liver egg burden in infected mice. Female IL-33^−/−^ and WT BALB/c mice (4–8 animals per group) were subcutaneously infected with 50 and 35 *S. mansoni* cercariae for 9 and 12 weeks, respectively, and sacrificed at 6, 9 and 12 wpi to determine the number of worm pairs and assess the number of liver tissue eggs. **a** Number of eggs per worm pair, **b** number of eggs per gram of liver tissue. Experiments were replicated at least three times. Data are representative of 2 independent experiments with similar results and are presented as mean with SEM. Groups were compared using unpaired two-tailed t-test with Welch’s correction, with statistical significance set at *P* < 0.05

**Additional file 2: Figure S2.** Oviposition in *S. mansoni* infection induces intestinal production of IL-25 and TSLP but not of IL-33. Female WT BALB/c mice (3 animals per time point) were subcutaneously infected with 50 and 35 *S. mansoni* cercariae for 9 and 12 weeks, respectively, and sacrificed weekly from week 0 (non-infected) to week 4, then every 2 weeks up to week 12 of infection. Small intestines were homogenized with the gentleMACS Octo Dissociator, and the cytokines were measured in the homogenate supernatants by ELISA. Data are presented as the mean with standard deviation. Cytokines were measured in only one mouse at 12 wpi

## Data Availability

All data generated or analyzed during this study are included in this published article and its Additional files.

## Abbreviations

BCA: Bicinchoninic acid
ESP: Excretory–secretory product
HBSS: Hank’s balanced salt solution
IL: Interleukin
ILC2: Group 2 innate lymphoid cell
M2: Alternatively activated macrophage
MLN: Mesenteric lymph node
NBF: Neutral buffered formalin
PBS: Phosphate buffered saline
*Sm*SEA: *Schistosoma mansoni* soluble egg antigens
Th2: T-helper 2 cell
TSLP: Thymic stromal lymphopoietin
wpi: Weeks post-infection
WT: Wild type
ω1: Omega-1
