# Supplementary figures and images for: Interleukin (IL)-33 is dispensable for Schistosoma mansoni worm maturation and the maintenance of egg-induced pathology in intestines of infected mice
Source: Parasit Vectors. 2021 Jan 22;14:70. doi: 10.1186/s13071-020-04561-w (PMC7821721; doi:10.1186/s13071-020-04561-w)

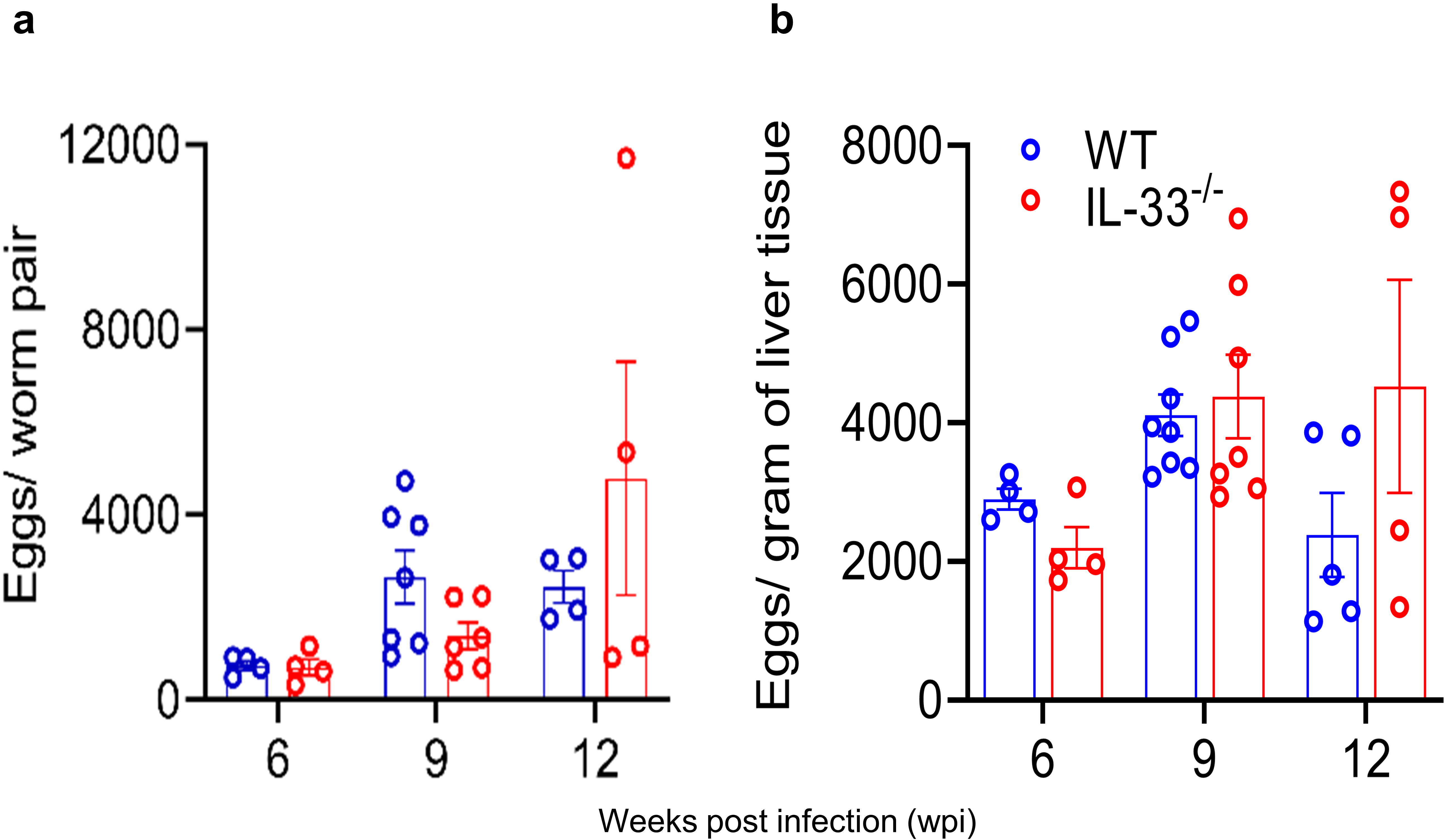

Supplement: Supplementary file 1 — Additional file 1: Figure S1. IL-33 deficiency does not affect the number of eggs produced per S. mansoni worm pair and the liver egg burden in infected mice. Female IL-33−/− and WT BALB/c mice (4–8 animals per group) were subcutaneously infected with 50 and 35 S. mansoni cercariae for 9 and 12 weeks, respectively, and sacrificed at 6, 9 and 12 wpi to determine the number of worm pairs and assess the number of liver tissue eggs. a Number of eggs per worm pair, b number of eggs per gram of liver tissue. Experiments were replicated at least three times. Data are representative of 2 independent experiments with similar results and are presented as mean with SEM. Groups were compared using unpaired two-tailed t-test with Welch’s correction, with statistical significance set at P < 0.05 [file 13071_2020_4561_MOESM1_ESM.tif]

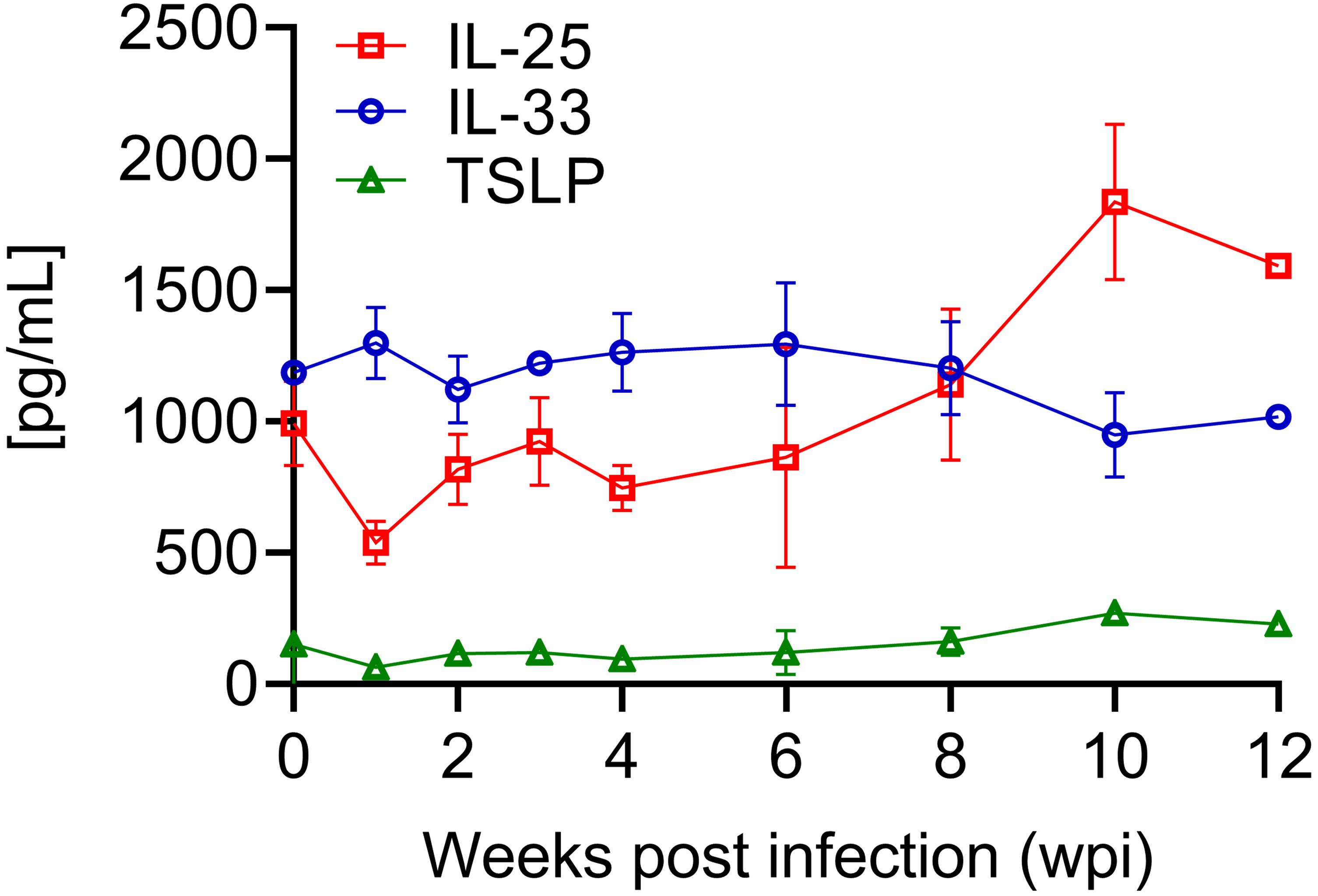

Supplement: Supplementary file 2 — Additional file 2: Figure S2. Oviposition in S. mansoni infection induces intestinal production of IL-25 and TSLP but not of IL-33. Female WT BALB/c mice (3 animals per time point) were subcutaneously infected with 50 and 35 S. mansoni cercariae for 9 and 12 weeks, respectively, and sacrificed weekly from week 0 (non-infected) to week 4, then every 2 weeks up to week 12 of infection. Small intestines were homogenized with the gentleMACS Octo Dissociator, and the cytokines were measured in the homogenate supernatants by ELISA. Data are presented as the mean with standard deviation. Cytokines were measured in only one mouse at 12 wpi [file 13071_2020_4561_MOESM2_ESM.tif]
